# Knee and ankle range of motion and spasticity from childhood into adulthood: a longitudinal cohort study of 3,223 individuals with cerebral palsy

**DOI:** 10.2340/17453674.2024.40606

**Published:** 2024-05-06

**Authors:** Erika CLOODT, Anna LINDGREN, Elisabet RODBY-BOUSQUET

**Affiliations:** 1 Department of Clinical Sciences Lund, Orthopaedics, Lund University, Lund; 2 Department of Research and Development, Region Kronoberg, Växjö; 3 Centre for Mathematical Sciences, Lund University, Lund; 4 Centre for Clinical Research Västerås, Uppsala University-Region Västmanland, Västerås, Sweden

## Abstract

**Background and purpose:**

Reduced range of motion (ROM) and spasticity are common secondary findings in cerebral palsy (CP) affecting gait, positioning, and everyday functioning. These impairments can change over time and lead to various needs for intervention. The aim of this study was to analyze the development path of the changes in hamstring length, knee extension, ankle dorsiflexion, and spasticity in hamstrings and gastrosoleus from childhood into adulthood in individuals with CP at the Gross Motor Function Classification System (GMFCS) levels I–V.

**Methods:**

A longitudinal cohort study was undertaken of 61,800 measurements in 3,223 individuals with CP, born 1990–2017 and followed for an average of 8.7 years (range 0–26). The age at examination varied between 0 and 30 years. The GMFCS levels I–V, goniometric measurements, and the modified Ashworth scale (MAS) were used for repeated assessments of motor function, ROM, and spasticity.

**Results:**

Throughout the follow-up period, knee extension and hamstring length exhibited a consistent decline across all individuals, with more pronounced decreases evident in those classified at GMFCS levels III–V. Ankle dorsiflexion demonstrated a gradual reduction from 15° to 5° (GMFCS I–IV) or 10° (GMFCS V). Spasticity levels in the hamstrings and gastrosoleus peaked between ages 5 and 7, showing a propensity to increase with higher GMFCS levels.

**Conclusion:**

Passive ROM continues to decrease to 30 years of age, most pronouncedly for knee extension. Conversely, spasticity reached its peak at a younger age, with a more notable occurrence observed in the gastrosoleus compared with the hamstrings. Less than 50% of individuals had spasticity corresponding to MAS 2–4 at any age.

Reduced range of motion (ROM) and spasticity are common secondary symptoms in cerebral palsy (CP) affecting gait, positioning, and everyday functioning [1]. Individuals with CP often have recurrent contact with healthcare during their entire life and interventions are decided based on clinical examinations in combination with goal setting and level of functioning. CP is a nonprogressive disorder; however, joint ROM, deformities, and spasticity causing pain and impaired function can worsen over time. Spasticity, contractures, and deformities are associated with pain and the prevalence of pain increases with age and severity of CP [2].

Systematic follow-up programs for CP have been created in several countries with the aim of detecting changes in musculoskeletal functioning and preventing severe complications such as hip dislocations, joint contractures, and scoliosis. In Sweden, the follow-up program, CPUP, has followed children with CP systematically since 1994. A study from 2005 showed a 25% reduction in orthopedic surgeries for contractures over a 10-year period [3].

Previous studies on ROM and spasticity have been either cross-sectional [1], up to 15 years of age [4], or focused on ambulant children [5], even though contractures and spasticity affect individuals at all Gross Motor Function Classification System (GMFCS) levels [2,6]. CP includes a heterogeneous group of neurological subtypes and knowledge of motor impairments according to age is mandatory. Therefore, being able to discriminate between levels within the diagnosis enables better prediction of development and directed treatment. This also holds significance in discussions involving individuals with CP and their families regarding the progression of contractures and spasticity. There is a need for studies including all GMFCS levels and analysis of ROM into adulthood [7].

The aim of this study was to analyze the development path of the changes in hamstring length, knee extension, ankle dorsiflexion, and spasticity in hamstrings and gastrosoleus from childhood into adulthood in individuals with CP at GMFCS levels I–V.

## Methods

**Figure 1:**
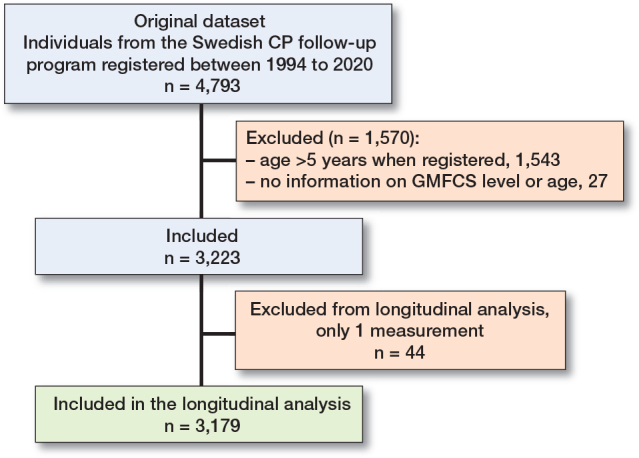
Flowchart of inclusion.

This was a longitudinal cohort study based on registry data from the Swedish CP follow-up program CPUP. This study includes all measurements reported in the registry from 1994 to 2020. CPUP includes > 95% of children with CP in Sweden and has a yearly reporting rate of 90–95%. Children with CP, or at risk of CP, born between 1990 and 2017 who had their first examination in the program before 5 years of age were included. Individuals included in the registry after 5 years of age were excluded. Only children with at least 2 examinations were included in the final analysis (Figure 1). In CPUP, individuals are followed up with regular multiprofessional examinations, at their local habilitation center, including gross and fine motor function, passive ROM, spasticity assessment, mobility, physical activity, and pain. The frequency of examinations depended on age and GMFCS level and varied between twice a year and every second year.

A physiotherapist classified each child’s gross motor function at each examination according to the GMFCS level. The most frequently scored GMFCS level was used for each child for the entire follow-up period. Passive ROM was measured using a goniometer in standardized positions with the individual lying supine on the examination table. Hamstring length was measured as the popliteal angle with the contralateral leg in full extension. The degrees were recorded as the angle between femur and tibia [8]. For example, a popliteal angle of 30° was recorded as the actual knee angle of 150° and used as a substitute measure to indicate the length of the hamstring in the CPUP registry. Knee extension was measured with the hip extended and ankle dorsiflexion with the knee extended. The ROM was rounded to the nearest 0° or 5° according to standard. The level of spasticity was rated in hamstrings and gastrosoleus in a supine position using the modified Ashworth scale (MAS). All assessments were performed by the child’s local team, with one examiner positioning the joint and the other measuring with a goniometer. Variables included in this study were age, sex, GMFCS level, unilateral/bilateral engagement, ROM, and spasticity. This longitudinal cohort study was designed and reported in accordance with the STROBE guidelines for cohort studies.

### Statistics

All legs were followed individually in the analysis. For children with unilateral CP, only the affected leg was included. Due to the low number of participants classified at GMFCS level II as the most common classification, GMFCS levels I–II were analyzed together. To model the non-linear change in ROM and spasticity, as observed also in previous study [4], we used linear splines, where the knot value was the same for all children with the same GMFCS level but with individual random slopes before and after the breakpoint. To analyze the development of spasticity, MAS was grouped into 2 categories, MAS 0–1 and MAS 2–4, and the probability of having MAS 2–4 in relation to age was analyzed using logistic regression with a piecewise linear function for the log-odds, with random effects in the same way as for the development of ROM. In both cases, the knot values were chosen using cross-validation. The 95% confidence limits for the mean development were calculated using parametric bootstrapping. All statistical analysis was conducted using R 3.6.1 (R Foundation for Statistical Computing, Vienna, Austria). Categorical variables were described in frequencies (n) and percentages (%).

### Ethics, funding, and potential conflicts of interest

This study was approved by the Swedish Ethical Review Authority (LU-433-99, Dnr 2020-04511) and permission was obtained to extract data from the CPUP registry. The study received funding from Stiftelsen för bistånd åt rörelsehindrade i Skåne, Stiftelsen Promobilia, and Region Kronoberg. The funding sources had no decision-making role or influence on the study design or results. The authors declare that they have no conflicts of interest. Complete disclosure of interest forms according to ICMJE are available on the article page, doi: 10.2340/17453674.2024.40606

## Results

Data from 3,223 children with CP (1,348 [42%] girls and 1,875 [58%] boys) were included (Figure 1). There were 61,800 assessments over a period of 26 years. The analysis was based on 5,673 legs followed for an average of 8.7 years (range 0–26 years). The distribution of GMFCS levels was 59% at level I, 1% at level II, 10% at level III, 14% at level IV, and 17% at level V. Nearly all individuals with unilateral CP were within level I (Table 1).

**Table 1:** Number of legs and involvement for children at GMFCS levels I–V

GMFCS level	Children n (%)	Legs n (%)	Involvement, legs, n (%)		Limb years (mean follow-up)
			Unilateral CP	All other subtypes	
I	1,893 (59)	3,034 (53)	752 (97)	2,282 (47)	25,985 (8.6)
II	25 (1.0)	43 (1.0)	7 (1.0)	36 (1.0)	292 (6.8)
III	312 (10)	611 (11)	13 (1.7)	598 (12)	5,472 (9.0)
IV	453 (14)	905 (16)	1 (0)	904 (18)	8,651 (9.6)
V	540 (17)	1,080 (19)	0 (0)	1,080 (22)	9,018 (8.4)
Total	3,223	5,673	773	4,900	49,417 (8.7)

GMFCS = Gross Motor Function Classification System.

**Figure 2:**
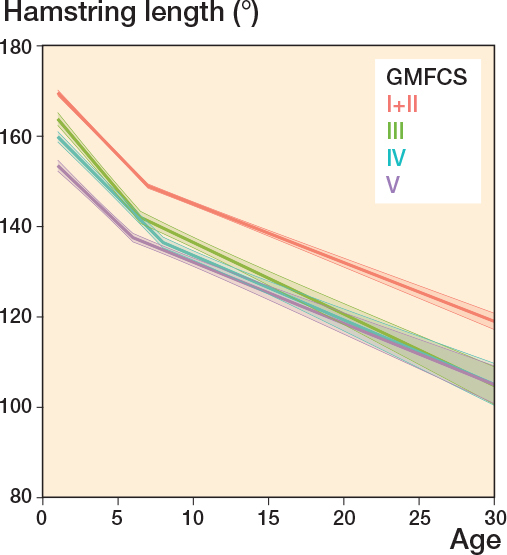
Hamstring length. Development of mean range of motion over age by GMFCS level. Piecewise random effects linear regression with 95% bootstrap confidence intervals.

Hamstring length decreased during the entire follow-up period for individuals at all GMFCS levels. The trend was similar for all GMFCS levels with a more rapid decrease until 7 years of age, followed by a slower decline through adolescence and adulthood (Figure 2). GMFCS levels III–V showed a median hamstring length of ≤ 130° from 12–18 years of age and for the remaining follow-up time (Table 2).

**Table 2:** Median range of motion (°) with (25th to 75th percentiles) or the percentage of children with modified Ashworth scale 2–4 by age and GMFCS level

GMFCS level Motion/measure	Age				
	0–5	6–11	12–17	18–23	24–31
I+II, n of legs	3,075	2,584	1,267	256	98
Hamstring length (°)	160 (150 to 170)	145 (140 to 155)	140 (130 to 150)	140 (130 to 150)	140 (130 to 150)
Knee extension (°)	0 (0 to 5)	0 (0 to 5)	0 (0 to 0)	0 (–5 to 0)	0 (–5 to 0)
Ankle dorsiflexion (°)	15 (10 to 20)	10 (5 to 15)	10 (0 to 15)	5 (0 to 10)	5 (0 to 10)
Hamstring spasticity (%)	3.8	4.5	5.4	11	16
Gastrosoleus spasticity (%)	27	23	19	19	23
III, n of legs	611	504	239	59	25
Hamstring length (°)	150 (140 to 160)	135 (130 to 150)	130 (120 to 140)	130 (115 to 140)	130 (120 to 145)
Knee extension (°)	0 (0 to 0)	0 (–5 to 0)	–10 (–20 to 0)	–10 (–20 to 0)	–15 (–20 to –5)
Ankle dorsiflexion (°)	15 (5 to 20)	10 (0 to 15)	10 (5 to 15)	10 (0 to 15)	5 (0 to 10)
Hamstring spasticity (%)	15	21	25	20	8.1
Gastrosoleus spasticity (%)	40	33	19	15	18
IV, n of legs	897	775	412	106	24
Hamstring length (°)	150 (140 to 160)	135 (125 to 150)	130 (120 to 140)	130 (120 to 140)	130 (115 to 131)
Knee extension (°)	0 (0 to 0)	–5 (–10 to 0)	–10 (–20 to 0)	–15 (–30 to –5)	–30 (–35 to –20)
Ankle dorsiflexion (°)	15 (5 to 20)	10 (0 to 15)	10 (0 to 20)	10 (0 to 15)	5 (0 to 10)
Hamstring spasticity (%)	23	24	28	28	21
Gastrosoleus spasticity (%)	46	36	23	39	32
V, n of legs	1,078	835	414	82	38
Hamstring length (°)	145 (135 to 155)	135 (125 to 145)	130 (115 to 140)	125 (115 to 130)	120 (110 to 125)
Knee extension (°)	0 (–5 to 0)	–5 (–15 to 0)	–15 (–30 to –5)	–20 (–30 to –10)	–25 (–40 to –15)
Ankle dorsiflexion (°)	15 (5 to 20)	10 (0 to 20)	10 (0 to 20)	15 (10 to 20)	10 (5 to 15)
Hamstring spasticity (%)	30	30	26	30	23
Gastrosoleus spasticity (%)	48	40	26	27	30

GMFCS = Gross Motor Function Classification System.

**Figure 3:**
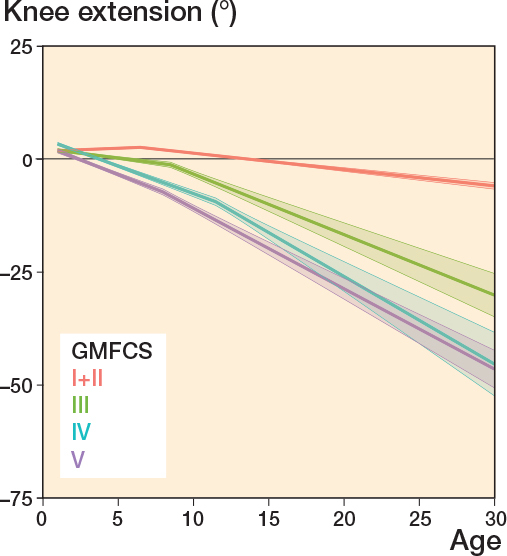
Knee extension, see Legend to Figure 2.

Knee extension remained unchanged during the entire follow-up period for those at GMFCS levels I+II with a median ROM of 0° in all age groups (Table 2), whereas the median knee extension decreased during follow-up for individuals in GMFCS levels III–V (Figure 3). Knee extension decreased from 0° in ages 0–6 years to a median value of –15° for GMFCS III, –30° for GMFCS IV, and –25° for GMFCS V in ages 24–31 years (Table 2).

**Figure 4:**
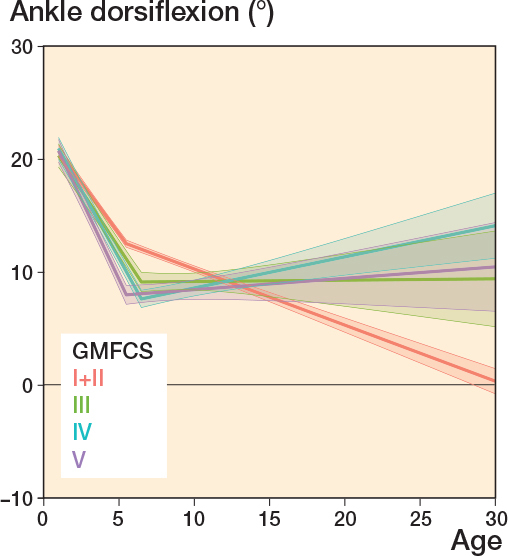
Ankle dorsiflexion, see Legend to Figure 2.

The median ankle dorsiflexion decreased slowly up to 24–31 years of age for all GMFCS levels (Figure 4) with a median ROM of 15° at ages 0–6 years to a median ROM of 5° (GMFCS I–IV) or 10° (GMFCS V) at ages 24–31 years. None of the GMFCS levels showed a median dorsiflexion under 0° at any age (Table 2).

**Figure 5:**
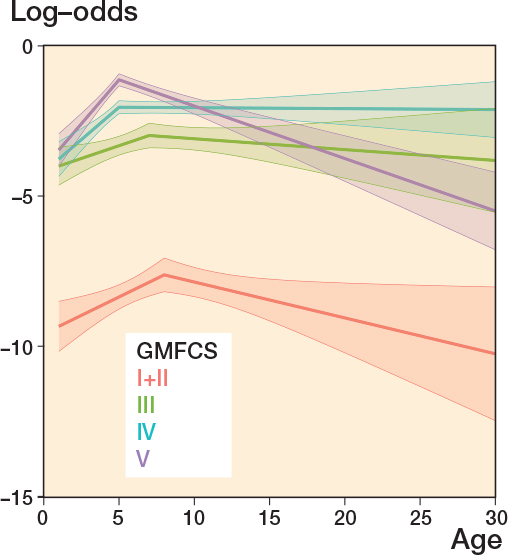
Hamstring spasticity ^a^. ^a^ Development of log-odds of modified Ashworth scale score 2–4 vs. 0–1 over age by GMFCS level. Log-odds = 0 means that the alternatives 2–4 and 0–1 are equally likely. Log-odds < 0 means that the alternative 2–4 is less likely than the alternative 0–1. Also see Legend to Figure 2.

The risk of hamstring spasticity was low during the entire follow-up period in GMFCS levels I+II. The risk of higher spasticity level peaked at 5–7 years of age for GMFCS levels III–V and then either remained unchanged in adulthood (GMFCS III and IV) or decreased slightly (GMFCS V) (Figure 5). The prevalence of hamstring spasticity of MAS 2–4 increased with higher GMFCS level; 3.8% of children at GMFCS levels I+II had a reported spasticity of MAS 2–4 at age 0–6 years compared with 30.4% of those at GMFCS level V. Less than 31% of individuals reported hamstring spasticity of MAS 2–4 at any age (Table 2).

**Figure 6:**
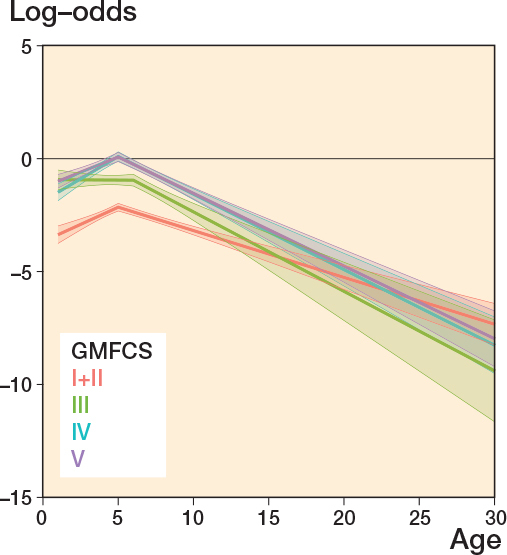
Gastrosoleus spasticity ^a^. ^a^ Development of log-odds of modified Ashworth scale score 2–4 vs. 0–1 over age by GMFCS level. Log-odds = 0 means that the alternatives 2–4 and 0–1 are equally likely. Log-odds < 0 means that the alternative 2–4 is less likely than the alternative 0–1. Also see Legend to Figure 2.

The likelihood of having a higher level of gastrosoleus spasticity (MAS 2–4) increased up to 5–6 years of age and then decreased during the entire follow-up period to adulthood for all GMFCS levels (Figure 6). Spasticity in gastrosoleus was more common than hamstring spasticity. The prevalence of gastrosoleus spasticity increased with GMFCS level; 27% of children in GMFCS levels I+II had a reported spasticity of MAS 2–4 at age 0–6 years compared with 48% of those in GMFCS level V. Less than 50% of individuals reported gastrosoleus spasticity of MAS 2–4 at any age (Table 2).

## Discussion

This study aimed to follow the development of hamstring length, knee extension, ankle dorsiflexion, and spasticity in hamstrings and gastrosoleus and is the first longitudinal study that has analyzed these changes from childhood to adulthood in CP at all GMFCS levels. We showed that the prevalence of reduced muscle length and higher spasticity level increased with higher GMFCS level. ROM decreased most rapidly before 10 years of age and the likelihood of having higher spasticity levels peaked at 5–7 years of age. Both hamstring length and knee extension continued to decrease from early childhood to adulthood, and we observed a clinically significant change in mean ROM from the youngest age group to adulthood. Ankle dorsiflexion remained more stable.

The median hamstring length continued to decrease for all GMFCS levels up to adulthood. At the age of 15 years, all GMFCS levels had a median hamstring ROM < 140°, which is almost identical to the findings from a Norwegian cohort study from 2020 [5]. Both that study and our study showed that the trend of changes in hamstring length was similar across GMFCS levels. Individuals with CP have reduced hamstring length compared with individuals without CP [7]. In a study of 140 healthy adults by Moon et al. (2017) [9], hamstring length (measured as the unilateral popliteal angle) was equal in all age groups (from 13 to > 51 years) and always ≥ 140°.

Compared with studies on individuals without CP [9,10], our findings show that lower limb ROM and muscle length are not only reduced in individuals with CP, but they also decrease with age. This result was especially visible for hamstring length and knee extension. Both these measures involve the knee joint and the hamstring muscles. Biopsies from hamstring muscles from individuals with CP have shown a muscle pathology with less than half as many satellite cells as typically developed muscles [11]. Satellite cells are responsible for muscle growth and, as the skeleton grows, muscles of children with CP might not grow as much as needed, resulting in highly stretched sarcomeres, smaller muscle belly, and long tendons [11,12]. This study shows that hamstring length decreases rapidly from an early age to a mean value of < 140° by the age of 10 years for those at GMFCS levels III–V. As the femur is the longest bone in the human body, it is likely that relative muscle shortening affects hamstrings in particular. Reduced hamstring length is strongly associated with knee contractures, whereas hamstring spasticity appears to have only a weak association [13]. In this study, knee extension also decreased from childhood to adulthood. Regarding mean ROM, all GMFCS levels had a median knee extension < 0° from 13 years of age.

Our results show that passive dorsiflexion decreased rapidly until the age of 5 years as analyzed by linear regression with a breakpoint. Comparing mean values across GMFCS groups, all groups except for level V continued to lose dorsiflexion during the entire follow-up period. A dorsiflexion of ~10° is often considered as a normal ROM [14]. However, passive dorsiflexion does not automatically correlate with gross motor performance [15]. In a 2014 study, ankle–foot orthoses were most commonly used in children at GMFCS levels IV and V and often with the intention to improve or maintain ankle dorsiflexion [16]. Compared with individuals without CP, the results of our study showed a reduced mean ROM in dorsiflexion in all age groups [9,10].

Our study shows that the likelihood of spasticity (rated as MAS level 2–4) peaked at 5–7 years of age for both the hamstrings and gastrosoleus muscle. This result agrees with a study on gastrocnemius spasticity from Sweden [4] and a Norwegian study of hamstring length and spasticity [5]. Individuals at GMFCS levels I+II had lower risk of spasticity (MAS 2–4) in the gastrosoleus muscle compared with those at higher GMFCS levels. Gastrosoleus is the most common location for botulinum toxin injections and this intervention is most frequently administered to children at GMFCS levels I+II [17]. In our study, the likelihood of MAS 2–4 in the hamstrings compared with MAS 0–1 was higher for those at GMFCS levels III–V. Children at GMFCS levels I+II had a low risk of spasticity (MAS 2–4) in hamstrings throughout the follow-up, which was similar to the Norwegian study [5]. Botulinum toxin injections for children at GMFCS levels III–V are most commonly given into the hamstrings [17]. It is important to note that individuals in this study might have received spasticity-reducing treatment during the follow-up period, which could have had a transient or lasting impact on their spasticity levels.

### Limitations

We did not separate unilateral and bilateral involvement in the analysis but included only the affected leg of children with unilateral CP. In this study, we did not specifically examine how different subtypes impact ROM and spasticity. Previous research has highlighted that subtypes are a less accurate predictor of ROM and function compared with GMFCS [18]. Our decision to include individuals who have undergone orthopedic surgeries, received spasticity-reducing medication, and undergone serial casting stems from the ubiquitous nature of interventions among individuals with cerebral palsy (CP) in Sweden. Given the use of various treatments such as training, orthoses, and surgeries, determining a “natural” developmental trajectory becomes intricate in longitudinal studies.

The actual length of the hamstring muscles is difficult to measure in the clinic because the muscles reach over 2 joints. The popliteal angle (PA) measurement is a commonly used method to assess hamstring length. It is possible that executing unilateral measurements might pose challenges for individuals with hip flexion contracture and potentially provide a misleading measurement of hamstring length in these individuals. However, in this study, such difficulty could potentially lead to a more favorable interpretation of their popliteal angle than is actually the case, and underestimate rather than overestimate the problem with reduced hamstring length. Spasticity can be challenging to differentiate from muscle stiffness, and the precise nature of spasticity remains not entirely elucidated [19]. The modified Ashworth scale has a relatively low reliability. However, the Ashworth and modified Ashworth scales, together with the Tardieu scale, are the most commonly used tools to measure spasticity in clinical practice [20]. To avoid overestimating spasticity levels, we chose to define spasticity as MAS 2–4 with more marked increase in muscle tone through most of the ROM.

The time between examinations is based on GMFCS levels and age according to CPUP and varies from twice a year to every second year, which means that the number of examinations differed and changes between examinations may not have been detected.

### Strengths

The strength of this study lies in its longitudinal design, which systematically follows a total population over a span of 26 years. This approach provides a robust framework for tracking long-term outcomes and understanding developmental trajectories.

Various interventions have evolved throughout the duration of the study period. All individuals in this study are included in a follow-up program with regular examinations and interventions.

### Conclusion

Passive ROM continues to decrease to 30 years of age, most pronouncedly for knee extension. Conversely, spasticity reaches its peak at a younger age, with a more notable occurrence observed in the gastrosoleus compared with the hamstrings. Less than 50% of individuals had spasticity corresponding to MAS 2–4 at any age.

## Notes

Study design, data collection, data analysis, and manuscript preparation: EC, AL, ERB.

The authors thank all the children and adults with CP and their families who participate in CPUP.

Handling co-editors: Ivan Hvid and Robin Christensen

*Acta* thanks Niels Wisbech Pedersen and Terje Terjesen for help with peer review of this manuscript.
